# Characterization of the complete chloroplast genome sequence of *Asparagus densiflorus ‘*Sprengeri*’* Huttleston 1970 (Asparagaceae)

**DOI:** 10.1080/23802359.2025.2582533

**Published:** 2025-11-03

**Authors:** Qiaoyu Zhang, Benteng Wang, Meng Mu, Xia Li, Yuan Lu, Xiaoxue Niu

**Affiliations:** ^a^ College of Horticulture, Xinyang Agriculture and Forestry University, Xinyang, P. R. China; ^b^ Asparagus Research Center, Weifang Academy of Agricultural Sciences, Shandong, P. R. China

**Keywords:** Asparagaceae, chloroplast genome, phylogenetic analyses

## Abstract

*Asparagus densiflorus ‘*Sprengeri*’* is an economically important ornamental and medicinal plant within the genus *Asparagus*. In this study, we sequenced and assembled its complete chloroplast (cp) genome using the Illumina NovaSeq platform. The circular cp genome is 157,116 bp in length with a GC content of 38%, comprising a large single-copy (LSC) region (85,327 bp), a small single-copy (SSC) region (18,677 bp), and two inverted repeat (IR) regions (26,556 bp each). The genome encodes 133 functional genes, including 87 protein-coding genes, 38 tRNA genes, and 8 rRNA genes. Phylogenetic analysis indicates that *A. densiflorus ‘*Sprengeri*’* is most closely related to *A. densiflorus ‘*Myers’ and *A. falcatus*, suggesting a shared evolutionary lineage. This study will not only shed light on *A. densiflorus ‘*Sprengeri*’’*s evolutionary position but also provide valuable chloroplast genomic information for future studies into the origins and diversification of the genus *Asparagus* and the Asparagaceae family.

## Introduction

*Asparagus* is reclassified as Asparagaceae through molecular phylogenetic studies (Fay, 2000; APG III/IV, Bremer et al. 2009; Chase et al. 2016), though originally classified in Liliaceae based on morphology (Bailey and Bailey 1976; Dahlgren et al. 1985). The genus includes about 300 species, native to southern Africa but now globally distributed (Norup et al. 2015). Several species are valued for medicinal (Chevallier, 1996; Leon and Lin 2017), culinary (Tardio et al. 2006), and ornamental uses (Walters, 1986).

*Asparagus densiflorus* demonstrates therapeutic potential against HIV and opportunistic infections (Gail, 2015), with bioactive extracts showing antimicrobial and anticancer properties (Mady et al. 2024). It also exhibits strong phytoremediation capacity, effectively degrading textile dyes and reducing wastewater toxicity (Watharkar et al. 2018).

Plastid genomes exhibit considerable architectural diversity across plant lineages. Although often depicted as circular molecules with a conserved quadripartite structure, their actual organization varies substantially—ranging in size from under 100 kbp to over 1,000 kbp, and existing as linear chromosomes, minicircles, or even being entirely lost in some species. This structural plasticity reflects processes of reductive evolution and adaptation, underscoring the dynamic nature of plastome evolution (de Vries and Archibald 2018).

Among the two recognized *A. densiflorus* cultivars (Huttleston, 1970), ‘Myers’ has been relatively well-studied (Ou et al. 2010; Wong et al. 2022; Sobhy et al. 2024), whereas *‘*Sprengeri*’* remains underreported, with many studies failing to specify cultivars. The cultivars are morphologically distinct, with *‘*Sprengeri*’* exhibiting characteristic 5 mm spines at the base of its scale-like leaves. Here, we present the first complete chloroplast genome assembly of *A. densiflorus ‘*Sprengeri*’*.

## Materials and methods

### Plant material collection and DNA extraction

**Figure 1.**
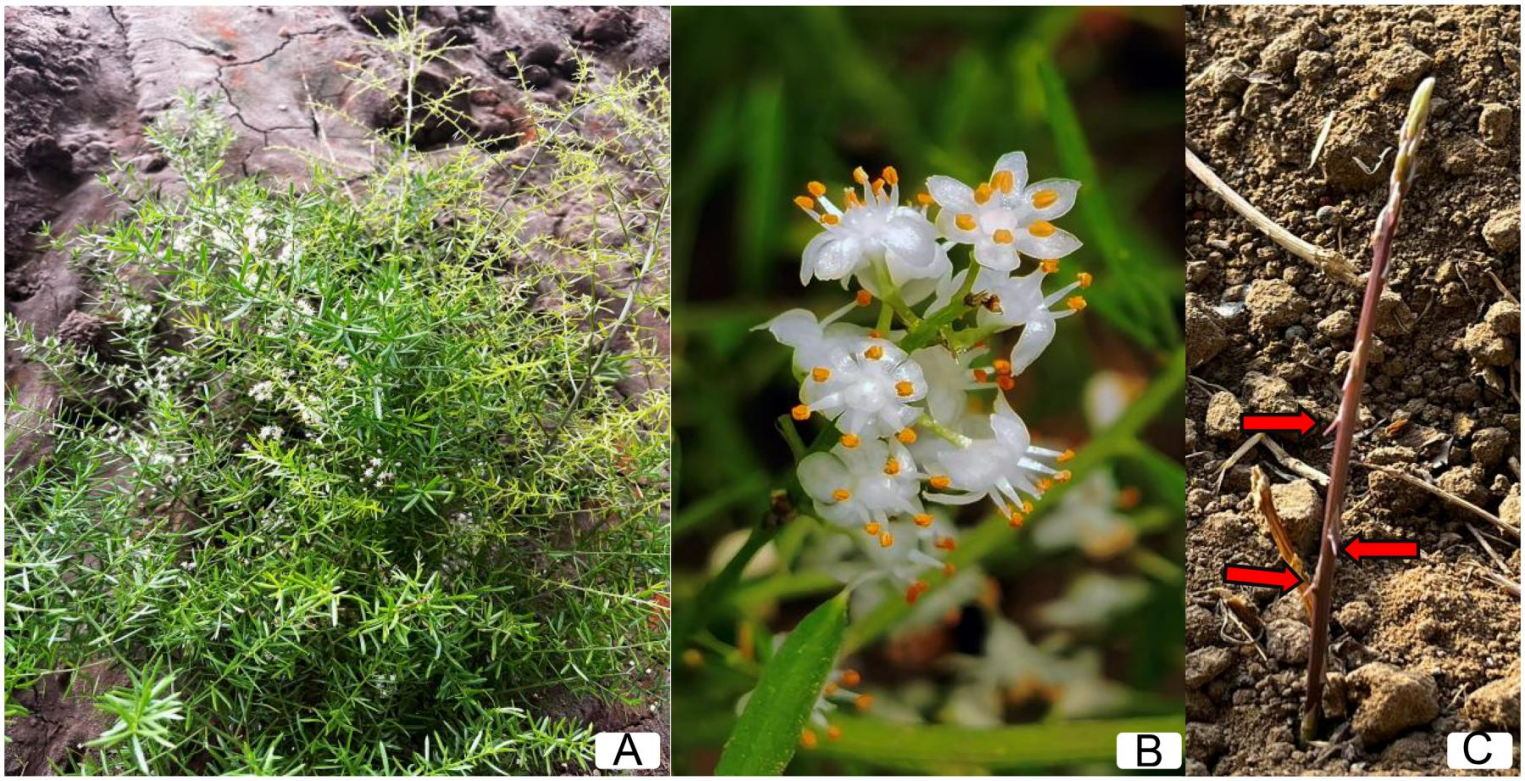
The specimen of the *A. densiflorus ‘*sprengeri*’*, (A)The whole plant, (B) The flowers, (C)The spins on the stem(red arrow marking). All the images were taken by Xiaoxue Niu. Main identifying traits: a semi-shrub to 1 m tall with flat, linear cladodes (leaf-like stems). Distinctive long, hard spines occur only at the base of scale-like leaves on main stems. Bears racemes of white flowers in summer, followed by red berries in autumn.

Plant samples of *A. densiflorus ‘*Sprengeri*’* were collected from Weifang, China (longitude 119°45′ E, latitude 36°69′ N) and authenticated by Dr. Xiaoxue Niu, a specialist in asparagus cultivation. Voucher specimens were planted at the Weifang Academy of Agricultural Sciences Modern Agricultural Science and Technology Demonstration Park (contact Xiaoxue Niu, apple.xiaoxue@163.com) under accession number YSZY202405 (Figure 1). Fresh leaves were packaged in thin foil, then frozen by liquid nitrogen for high throughput sequencing. Genomic DNA was extracted using a DNA easy Plant Mini Kit (Qiagen Co., Hilden, Germany) following the manufacture’s instructions. NanoDrop2000C spectrophotometry and electrophoresis in 1% (w/v) agarose gel were used to detect the concentration and integration of the total DNA, respectively.

### Sequencing, assembly, and annotation

Extracted DNA was fragmented to an average size of approximately 400 bp using CovarisM220 (Gene Company Limited, China) for paired-end library construction. Paired-end library was constructed using NEXTFLEX® Rapid DNA-Seq (BiooScientific, Austin, TX, USA). Adapters containing the full complement of sequencing primer hybridization sites were ligated to the blunt end of fragments. Paired-end sequencing was performed on Illumina NovaSeq platform (Illumina Inc., San Diego, CA, USA) at Majorbio Bio-Pharm Technology Co., Ltd. (Shanghai, China). Raw reads were quality controlled with Trimmomatic and Fast QC software (https://www.bio- informatics.babraham.ac.uk/projects/fastqc). Clean reads were then assembled using GetOrganelle with parameter ‘-R10 -k 21, 45, 65, 85, 105, 121 -Fembplant_pt’ (Jin et al. 2020).

The annotation of the *A. densiflorus ‘*Sprengeri*’* chloroplast genome was performed using CPGAVAS2 (http://www.cpgavas2) (Shi et al. 2019), with reference to the annotation of *Asparagus densiflorus* voucher Yang and Li 001(OQ851323.1), and manually curated using Apollo (Misra and Harris, 2005). The overall features of the *A. densiflorus ‘*Sprengeri*’* chloroplast genome were visualized using CPGview (Liu et al. 2023).

MISA (Beier et al. 2017) was utilized to detect simple sequence repeats (SSRs), including mono-, di-, tri-, tetra-, penta-, and hexa-nucleotides, with minimum occurrences of 10, 5, 4, 3, 3, and 3, respectively.

### Phylogenetic tree construction

A total of 29 whole chloroplast genomes from various *Asparagus* species were retrieved from the NCBI database for phylogenetic analysis, including *A. officinalis* (LN896356.1), *A. filicinus* (MK920078.1), *A. setaceus* (MK950153.1), *A. longiflorus* (OQ851313.1) and so on. *Cordyline indivisa* (KX822776) and *Eustrephus latifolius* (KM233639) were used as outgroup (Supplemental Table 1). These genomes were aligned using MAFFT (Katoh et al. 2002), and the phylogenetic tree was constructed using RaxML-ng after determining the best-fit model for phylogenetic inference (Minh et al. 2020). The resulting tree was visualized using iTOL (https://itol.embl.de/).

## Results

**Figure 2.**
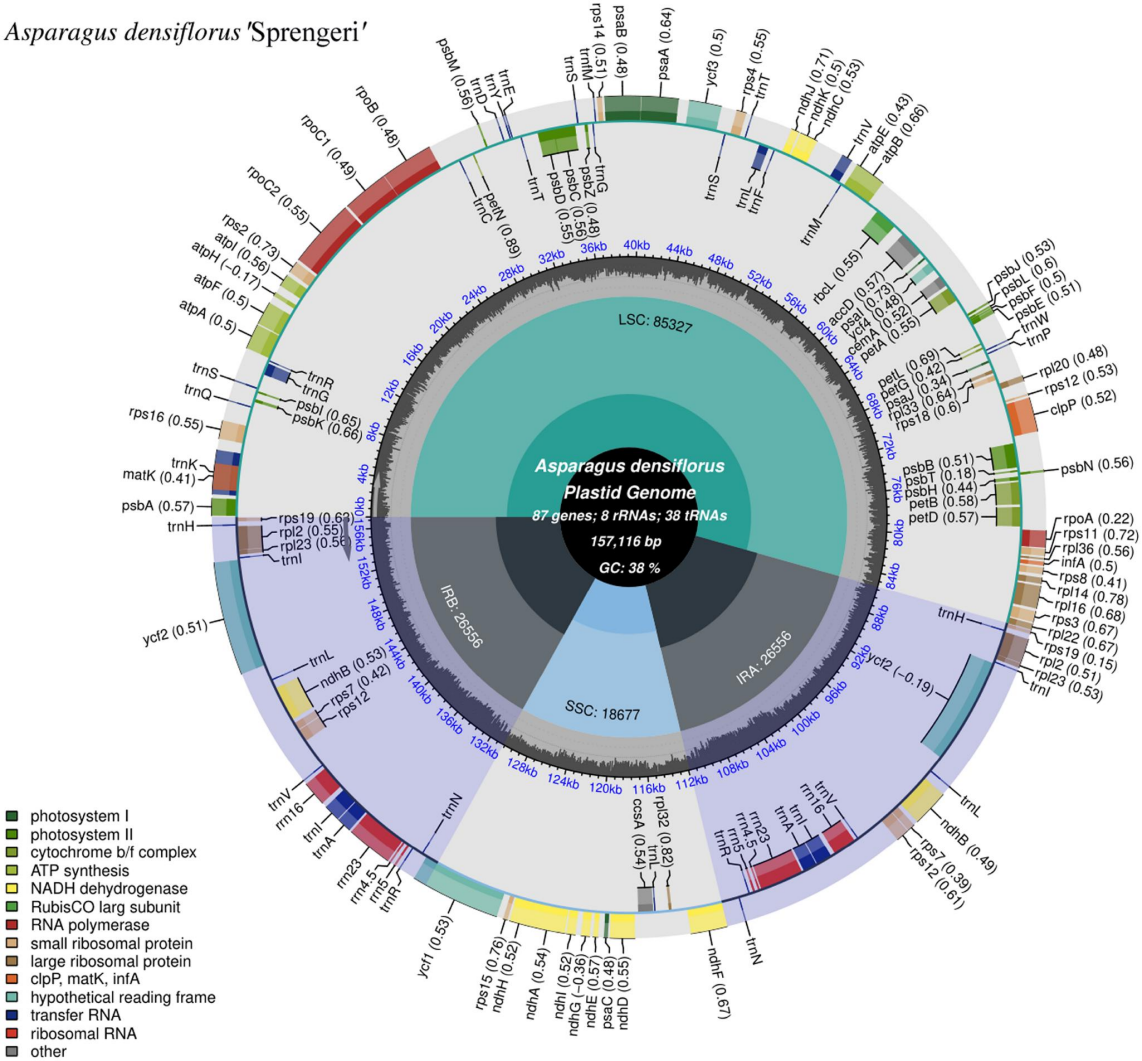
The complete chloroplast map of *A. densiflorus ‘*sprengeri*’*, which was generated by CPGview. LSC, SSC, and IRs (IRa and IRb) with their length are represented on the first circle. The second circle showed the GC ratio in dark gray. The outermost circle indicated gene names color-coded by their functional classification. The transcription directions for the inner and outer genes were clockwise and anticlockwise, respectively. The functional classification of the genes was shown in the bottom left corner. The optional codon usage bias was displayed in the parenthesis after the gene name.

A total of 300,747 paired reads were assembled to the complete chloroplast genome of *A. densiflorus ‘*Sprengeri*’*, revealing the average coverage depth was 2500.71× (Supplemental Figure 1). The complete chloroplast genome of *A. densiflorus ‘*Sprengeri*’* had been submitted to GenBank under the accession number PV658263. The genome exhibited a typical quadripartite structure (Figure 2) spanning 157,116 bp in total length. The large single copy (LSC) region comprised 85,327 bp, the small single copy (SSC) region comprised 18,677 bp, and a pair of inverted repeat (IR) regions separated the LSC and SSC, each region spanning 26,556 bp. The chloroplast genome encoded 133 (113 genes were unique) complete genes, including 87 (79 were unique) protein-coding genes, 38 (30 were unique) tRNA genes and 8 (4 were unique) rRNA genes (Supplemental Table 2). The average GC content was 38%. The schematic map of the cis-splicing genes in *A. densiflorus ‘*Sprengeri*’* chloroplast genome were showing in Supplemental Figure 2. Trans-splicing gene *rps12* had three unique exons. Two of them(exon2 and exon3) were duplicated as they were located in the IR regions (Supplemental Figure 3).

Annotation of the chloroplast genome identified a total of 56 SSRs, predominantly consisting of mono-nucleotide repeats (51; 91.1%), di-nucleotide repeats (4; 7.1%) and tri-nucleotide repeats (1; 1.8%) were very low (Supplemental Table 3 and Supplemental Figure 4).

**Figure 3.**
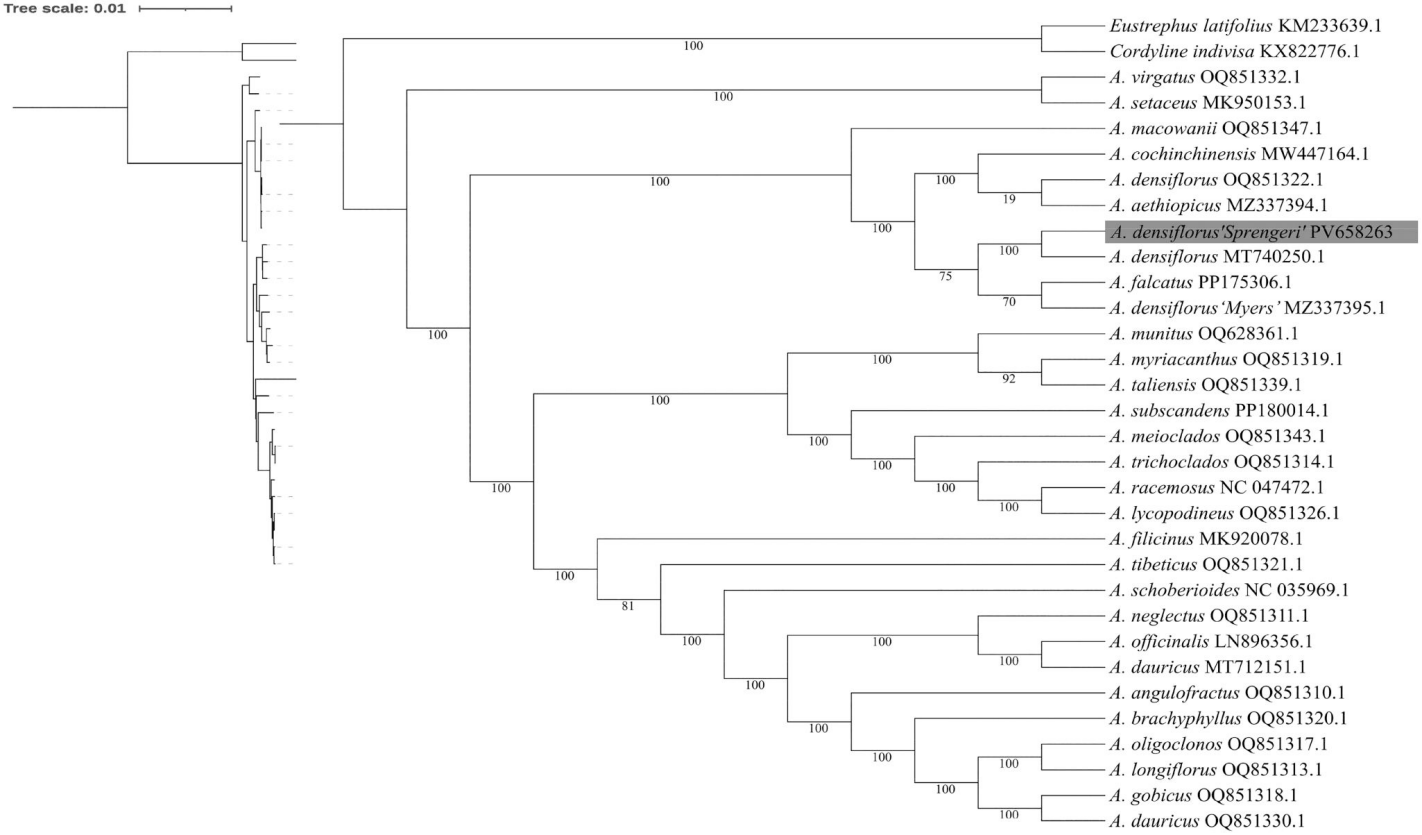
A maximum-likelihood (ML) based phylogenetic tree of *A. densiflorus ‘*sprengeri*’* and related *asparagus* species. The numbers on each node indicated the bootstrap support values. The overall phylogeny of all the species and outgroup displayed on the left. A detailed phylogenetic relationship of those species was presented on the right and as illustrated in Supplemental Table 1. *A. densiflorus ‘*sprengeri*’* (PV658263) was marked in bold. The following sequences were used: *A. officinals*(LN896356)(Li Q, 2019), *A. schoberioides* (NC035969)(Li Q, 2019), *A. filicinus* (MK920078)(Li Q, 2019), *A. setaceus* (MK_950153(Li JR, 2019), *A. densiflorus* (MT740250)(zhang R, 2022), *A. racemosus* (NC047472)(zhang R, 2022), *A. dauricus* (MT712151)(Sheng W, 2022), *A. cochinchinensis*(MW447164)(Sheng W, 2022), *A. taliensis* (OQ851339)(Xie P, 2025), *A. myriacanthus* (OQ851319)(Xie P, 2025), *A. lycopodineus* (OQ851326)(Xie P, 2025), *A. trichoclados* (OQ851314)(Xie P, 2025), *A. meioclados* (OQ851343)(Xie P, 2025), *A. tibeticus* (OQ851321)(Xie P, 2025), *A. neglectus*(OQ851311)(Xie P, 2025), *A. angulofractus* (OQ851310)(Xie P, 2025), *A. brachyphyllus* (OQ851320) (Xie P, 2025), *A. gobicus* (OQ851318)(Xie P, 2025), *A. dauricus* (OQ851330)(Xie P, 2025), *A. longiflorus* (OQ851313)(Xie P, 2025), *A. oligoclonos* (OQ851317)(Xie P, 2025), *A. densiflorus* (OQ851322)(Xie P, 2025), *A. macowanii* (OQ851347)(Xie P, 2025), *A. virgatus* (OQ851332)(Xie P, 2025), *A. falcatus* (PP175306)(Xie P, 2025), *A. munitus* (OQ628361)(Xie P, 2025), *A. subscandens* (PP180014)(Xie P, 2025), *A. aethiopicus* (MZ337394)(wong KH, 2022), *A. densiflorus ‘*myers*’* (MZ337395)(wong KH, 2022). *Cordyline indivisa* (KX822776) and *eustrephus latifolius* (KM233639) were used as outgroup.

To elucidate the phylogenetic position of *A. densiflorus ‘*Sprengeri*’* within *Asparagus*, we constructed a maximum likelihood tree using chloroplast genomes from 29 *Asparagus* species, including three *A. densiflorus* accessions from distinct sources. Phylogenetic analysis (Figure 3) revealed that *A. densiflorus ‘*Sprengeri*’* clusters closely with *A. densiflorus* (MT740250.1), *A. falcatus* (PP175306.1) and *A. densiflorus ‘*Myers*’*(MZ337395.1), indicating distinct evolutionary relationships among these taxa.

## Discussion and conclusions

This study presents the first complete chloroplast genome assembly of *A. densiflorus ‘*Sprengeri*’*, offering a valuable genomic resource for future phylogenetic and comparative analyses within the genus. Our phylogenetic reconstruction incorporated all three reported *A. densiflorus* accessions, revealing their consistent placement. Notably, one previously reported *A. densiflorus* (MT 740250.1)(Zhang R, 2022) formed a distinct subclade with our assembly, strongly supporting its identification as *A. densiflorus ‘*Sprengeri*’*. However, the remaining two *A. densiflorus* accessions exhibited divergent clustering patterns, each grouping separately with different *Asparagus* species.

Of particular interest is the observation that the *A. densiflorus* accession (OQ851322.1) reported by Xie (2025) neither clustered with the *A. densiflorus ‘*Sprengeri*’* lineage (represented by MT740250.1 and our data) nor with the *A. densiflorus ‘*Myers’ accession (MZ337395) identified by Wong (2022). This phylogenetic discordance suggests potential taxonomic complexities that warrant further investigation. Resolution of these discrepancies will likely require broader sampling of *Asparagus* chloroplast genomes as they become available.

It is also noteworthy that the inverted repeats (IRs) are unusual because the *YCF1* protein-coding gene is almost entirely absent from them, with only a minimal portion located within this region(IRb)(Figure 2). What makes this structural deviation notable is that *YCF1* is an essential protein (Drescher et al. 2000), characterized by N-terminal sequences that have been highly conserved over more than 700 million years of streptophyte evolution (de Vries et al. 2017).

This study presents the assembly of the chloroplast genome of A. densiflorus *‘*Sprengeri*’* using short-read data. The genome displays a typical quadripartite structure, akin to other members of the Asparagus genus. Certain distinctive features identified herein merit in-depth investigation.

## Supplementary Material

Supplemental Table.xls

Supplemental Figure.doc

## Data Availability

The genome sequence data that support the findings of this study are openly available in GenBank of NCBI at https://www.ncbi.nlm.nih.gov/ under the accession number PV658263. The associated BioProject, BioSample, and SRA numbers are PRJNA1265610, SAMN48602504, and SRR33676246, respectively.
