# Supplementary material for: Characterization of the complete chloroplast genome sequence of Asparagus densiflorus ‘Sprengeri’ Huttleston 1970 (Asparagaceae)
Source: Mitochondrial DNA B Resour. 2025 Nov 3;10(12):1088–92. doi: 10.1080/23802359.2025.2582533 (PMC12751095; doi:10.1080/23802359.2025.2582533)
Supplement: Supplemental Figure.doc [file TMDN_A_2582533_SM7673.doc]

Title:

**Characterization of the complete chloroplast genome sequence of *Asparagus densiflorus* 'Sprengeri' Huttleston 1970（Asparagaceae）**

Authors:

Qiaoyu Zhang^1^, Benteng Wang^1^, Meng Mu^2^, Xia Li^2^, Yuan Lu^2^, Xiaoxue Niu^2＊^

Author affiliation:

^1^College of Horticulture, Xinyang Agricultural and Forestry University, Xinyang, P. R. China

^2^Asparagus Research Center, Weifang Academy of Agricultural Science, Shandong, P. R. China

***Corresponding Author:**

Xiaoxue Niu:

No. 1921 Shengli East Street, Weifang, Shandong Province, 261071, P. R. China

Email address: apple.xiaoxue@163.com[;](mailto:wangcs@xyafu.edu.cn;) Phone:+86-15065678197

**Emails：**

Qiaoyu Zhang: [qiaoyu306@126.com;](mailto:qiaoyu306@126.com;)

Benteng Wang: [wangwent22@126.com;](mailto:406742883@qq.com;)

Meng Mu: [feng689@163.com;](mailto:wangcs@xyafu.edu.cn;)

Xia Li: [scaulxyl@163.com;](mailto:bailingm@163.com;)

Yuan Lu: lyuan2012@163.com


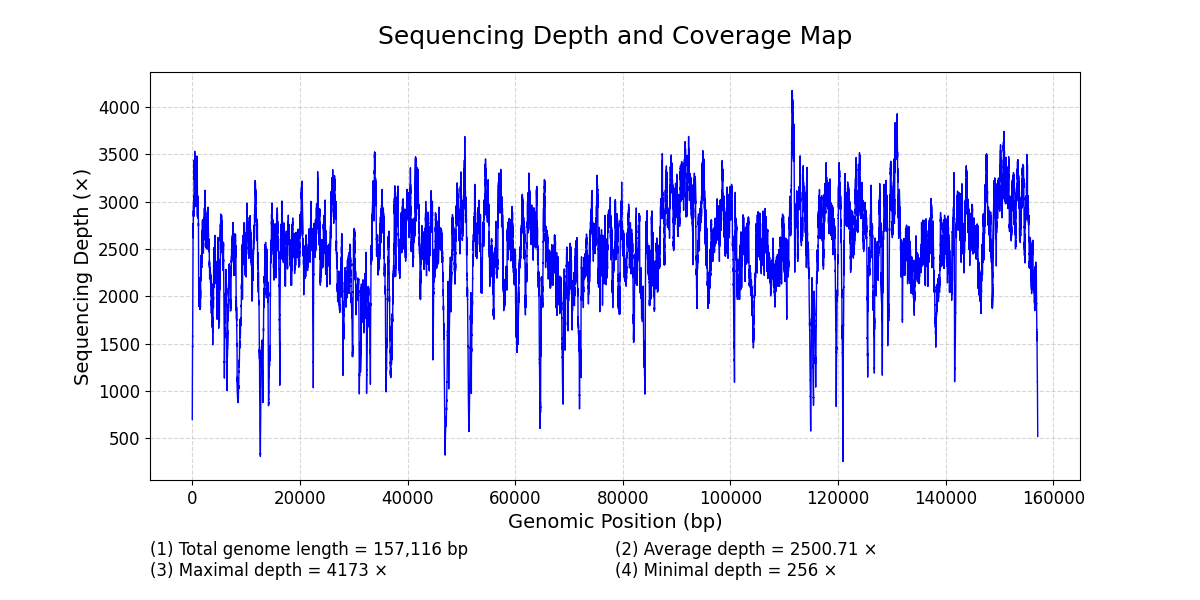


Supplementary Figure 1. Overall coverage and depth of the chloroplast genome assembly of *A. densiflorus* 'Sprengeri'. X-axis indicates the location of bases in the genome; Y-axis indicates the sequencing depth of each base in the genome.


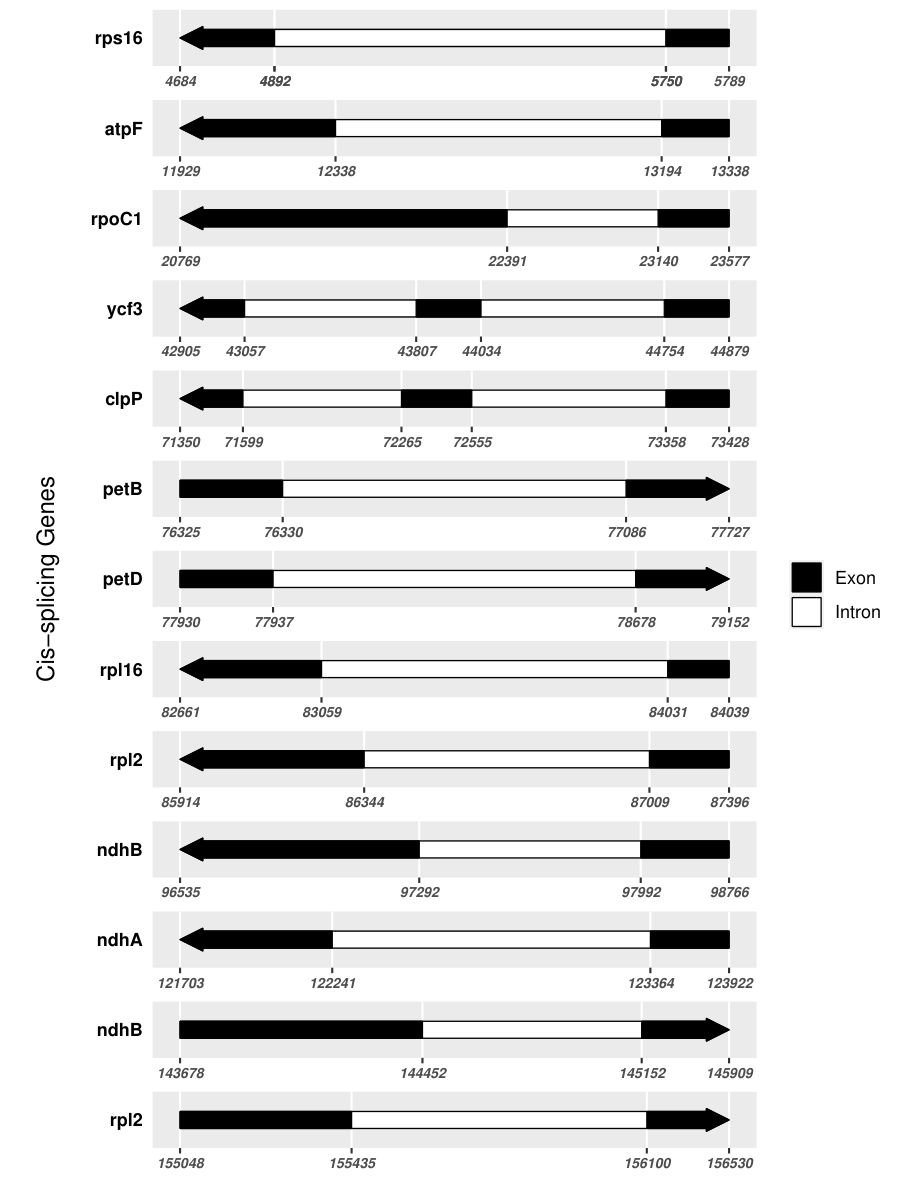


**Supplemental Figure 2. Schematic map of the cis-splicing genes in *A. densiflorus* 'Sprengeri' chloroplast genome.** The genes are arranged from top to bottom based on their order on the chloroplast genome. The gene names are shown on the left, and the gene structures are on the right. The exons are shown in black; the introns are shown in white. The arrow indicates the sense direction of the gene.


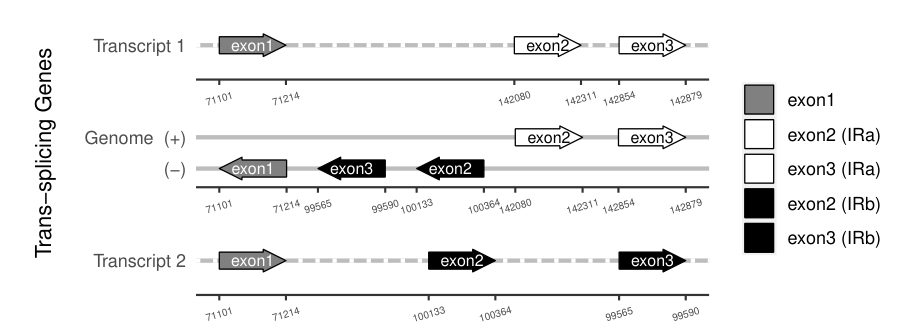


**Supplemental Figure 3. Schematic map of the trans-splicing gene *rps12* in the chloroplast genome of *A. densiflorus* 'Sprengeri'.** It has three unique exons. Two of them(exon2 and exon3) are duplicated as they are located in the IR regions.


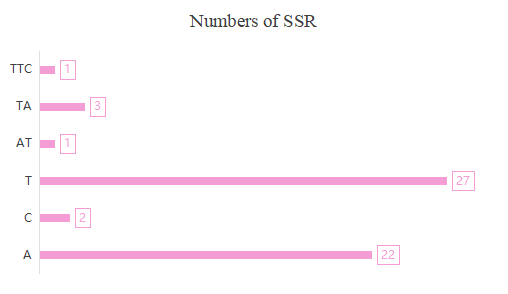


**Supplemental Figure 4. SSR sequence units identified in chloroplast genome of *A. densiflorus* 'Sprengeri'.** SSRs were characterized using MISA.
